# Phosphoprotein Profile of Rice (*Oryza sativa* L.) Seedlings under Osmotic Stress after Pretreatment with Chitosan

**DOI:** 10.3390/plants11202729

**Published:** 2022-10-15

**Authors:** Wasinee Pongprayoon, Atikorn Panya, Janthima Jaresitthikunchai, Narumon Phaonakrop, Sittiruk Roytrakul

**Affiliations:** 1 Department of Biology, Faculty of Science, Burapha University, 169 Longhaad Bangsaen Rd, Saensook, Mueang, Chonburi 20131, Thailand; 2 Functional Ingredients and Food Innovation Research Group, National Center for Genetic Engineering and Biotechnology (BIOTEC), 113 Thailand Science Park, Phaholyothin Rd., Klong Luang, Pathum Thani 12120, Thailand

**Keywords:** phosphoproteomics, chitosan, osmotic stress, Khao Dawk Mali 105, Leung Pratew 123, rice, LC-MS/MS

## Abstract

This study aims to identify novel chitosan (CTS)-responsive phosphoproteins in Leung Pratew 123 (LPT123) and Khao Dawk Mali 105 (KDML105) as drought-sensitive rice cultivars and differences in the CTS response. Rice seeds were soaked in CTS solution before germination, and 2- and 4-week-old rice seedlings sprayed with CTS before osmotic stress comprised the following four groups: (1) seedlings treated with distilled water; (2) seedlings treated with CTS; (3) seedlings pretreated with distilled water and subjected to osmotic stress; and (4) seedlings pretreated with CTS and subjected to osmotic stress. Phosphoproteins of leaf tissues were enriched using immobilized metal affinity chromatography (IMAC) before tryptic digestion and analysis via LC-MS. Phosphoprotein profiling analyses led to the identification of 4721 phosphoproteins representing 1052 and 1040 unique phosphoproteins in the LPT123 and KDML105 seedlings, respectively. In response to CTS pretreatment before osmotic stress, 22 differently expressed proteins were discovered, of which 10 and 12 were identified in the LPT123 and KDML105, respectively. These proteins are typically involved in signaling, transport, protein folding, protein degradation, and metabolism. This study provides fruitful data to understand the signal transduction mechanisms of rice seedlings pretreated with CTS before exposure to osmotic stress.

## 1. Introduction

Drought represents one of the most severe environmental factors that significantly limits the growth and productivity of plants. Because of the growing human population worldwide, increased irrigation demand, climate change, drought, and water deficits are expected to impact future agricultural practices [1]. Over the last decade, there has been a global effort to find alternative plant growth enhancers with fewer adverse environmental or human health effects to replace synthetic compounds and agrichemicals. Chitosan (CTS) has attracted attention as a potential plant protectant that is ideal for use in sustainable agriculture because of its environmental friendliness and natural biodegradability. The physicochemical properties of CTS have resulted in its application to food and nutrition, biotechnology, pharmaceuticals, agriculture, and environmental protection. CTS is a de-*N*-acetylated form of chitin, which is a copolymer comprised of randomly distributed β-1, 4-linked D-glucosamine (deacetylated) and *N*-acetyl-D-glucosamine (acetylated) units [2]. Chitin is also found as an organized crystalline microfibril that is a structural component of arthropod exoskeletons, fungal and yeast cell walls, and the shells of crustaceans, such as crabs and shrimp [3].

In agriculture, the application of CTS to plants as a potential natural polymer induced drought resistance through improved water-use efficiency in pepper (*Capsicum* sp.) leaves by 26%–43% [4]. In grapevine (*Vitis vinifera* L.) stems, a CTS concentration of 1.0% (*w/v*) induced drought tolerance by maintaining chlorophyll content under drought stress [5]. In other plants, a foliar application of CTS improved both growth and yield parameters under drought stress and unstressed conditions in cowpea (*Vigna unguiculata* (L.) Walp.) [6]. Pretreatment with CTS containing Hoagland’s solution prior to drought stress increased the production of stress-responsive metabolites in white clover (*Trifolium repens* L.) [7]. CTS application by spraying before the flowering stage resulted in an increase in flowering by 50% with full bloom and reduced the negative effects of drought stress in *Thymus daenensis* Celak. [8]. Spraying the foliar with CTS before flowering enhanced plant growth in sweet basil (*Ocimum ciliatum* L. and *O. basilicum* L.) [9] and rice (*Oryza sativa* L.) [10]. The mechanism through which CTS influences plant response, however, is not entirely understood. Nonetheless, it is thought that CTS initiates a complicated signaling cascade that results in changes in stress response and enhanced plant growth [11]. Reactive oxygen species (ROS), H_2_O_2_, Ca^2+^, nitric oxide (NO), and plant hormones (jasmonic acid, abscisic acid, and ethylene) have been identified as signaling molecules that respond to CTS and play an important role in many signaling processes [12].

In recent years, metabolome studies have suggested that CTS is involved in resistance to drought stress by promoting the accumulation of sugars, sugar alcohols, amino acids, organic acids, and other metabolites (ascorbate, glutathione, flavonoids, putrescine, and spermidine) [13]. These compounds are associated with osmotic adjustment, antioxidant defense, stress signaling, and energy metabolism during stress conditions. Transcriptome studies revealed that genes associated with the metabolism of amino acids, carbohydrates, carbon, photosynthesis, and plant hormones were significantly increased by CTS during osmotic stress [14]. Moreover, at the proteome level, rice leaf proteins were found in three central biochemical networks including photosynthesis, carbohydrate metabolism, and redox homeostasis. The genes positively coexpressed with CTS-responsive proteins were localized in chloroplasts, indicating that CTS enhanced the growth of rice seedlings through multiple complex networks between the nucleus and the chloroplasts [15]. Protein phosphorylation is a posttranslational modification of proteins that is considered important to many cellular processes, including signal transduction, development, metabolism, transcription, translation, and degradation [16]. To analyze the entire repertoire of cellular phosphoproteins or the phosphoproteome, several techniques have been developed to specifically enrich phosphoproteins coupled with LC-Mass spectra (MS)/MS analysis [17], which facilitates the detection of changes in low-abundance phosphoproteins. Large-scale phosphoproteomic analyses have been proposed for studies on plant growth, development, and stress defense in many plants [1]. However, there is limited information regarding phosphorylation modifications in response to CTS induction and osmotic stress in the rice genotype. 

Rice (*O. sativa* L.) is the most important staple food for more than half of the world’s population. However, abiotic stressors such as drought and high salinity affect rice growth and productivity. In this study, phosphoproteomes of drought-sensitive rice cultivars, Leung Pratew 123 (LPT123) and Khao Dawk Mali 105 (KDML105), were investigated. LPT123 and KDML105 cultivars were reported to respond to CTS pretreatment before exposure to osmotic stress [18,19]. However, limited studies report the function of CTS in improving drought resistance and CTS-induced changes in phosphoproteins in LPT123. Additionally, in the current research, LPT123 and KDML105 demonstrated different responses to CTS-induced resistance to osmotic stress in terms of physiological changes, providing a suitable model to study the response of the phosphoproteins at post-translational level to CTS induction. To evaluate the potential signal transduction mechanisms of CTS-induced phosphoproteins against osmotic stress, a gel-free quantitative phosphoproteomics approach was conducted. Therefore, the present study aimed to investigate rice seedlings potentially triggered by CTS under initiation and subjected to osmotic stress. This presumption implies substantial posttranslational phosphoprotein alterations in response to CTS under osmotic stress. The novel findings of this study provide new insights into understanding the regulatory mechanisms underlying the action of CTS-regulated resistance to osmotic stress in rice genotypes.

## 2. Results

### 2.1. Growth and Photosynthetic Pigments Induced by CTS Treatment and Osmotic Stress 

**Figure 1:**
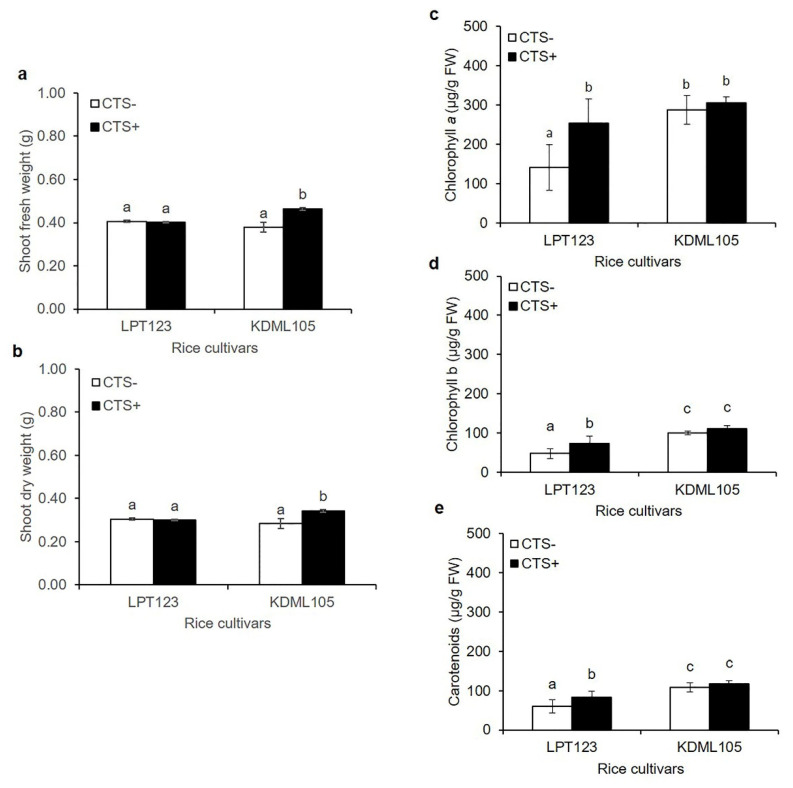
The effects of CTS treatment on seeds and seedlings of the LPT123 and KDML105 rice cultivars treated with and without CTS under osmotic stress for seven days showed shoot fresh weight (**a**) shoot dry weight; (**b**) chlorophyll *a*; (**c**) chlorophyll *b*; (**d**) and carotenoids; (**e**). Data are represented as mean ± SD, derived from 4 independent repeats. Different lowercase letters indicate significant differences, *p* < 0.05, ANOVA followed by Duncan’s multiple range test (DMRT).

The effect of chitosan (CTS) pretreatment before exposure to osmotic stress for 7 days was investigated in rice seedlings. CTS was applied via seed soaking and foliar spraying, as indicated in the materials. The CTS did not affect the shoot fresh weight (SFW) and shoot dry weight (SDW) of Leung Pratew 123 (LPT123) cultivar compared to the water treated seedlings. In contrast, CTS could enhance the SFW and SDW of the Khao Dawk Mali 105 (KDML105) cultivar by 1.3- and 1.2-fold, respectively (Figure 1a,b). After seven days of osmotic stress, CTS treatment significantly increased the chlorophyll (Chl) and carotenoid concentrations in the LPT123 cultivar. The Chl a, Chl b, and carotenoid contents of chitosan-treated plants were significantly increased by 1.8-, 1.5-, and 1.4-fold, respectively, compared to the control plants. In contrast, the CTS-treated KDML105 rice could maintain elevated photosynthetic pigment levels after osmotic stress (Figure 1c–e).

### 2.2. Differentially Expressed Proteins (DEPs) and Functional Classification

**Figure 2:**
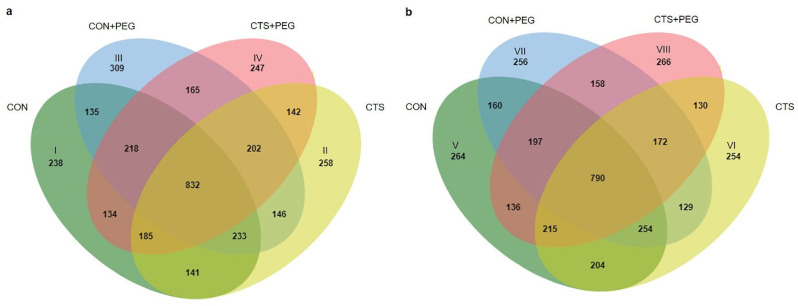
Venn diagrams showed the numbers of phosphoproteins in LPT123 (**a**) and KDML105; (**b**) cultivars. Differentially expressed phosphoproteins (DEPs) were retrieved among the four different treatment groups. CON (I and V), CTS (II and VI), CON + PEG (III and VII), and CTS + PEG (IV and VIII).

Leaf tissues from rice seedlings pretreated with CTS before exposure to osmotic stress were harvested. The four different treatments were as follows: (1) CON, seedlings treated with distilled water; (2) CTS, seedlings treated with CTS; (3) CON + PEG, seedlings pretreated with distilled water followed by PEG6000; and (4) CTS + PEG, seedlings pretreated with CTS followed by PEG6000. Total proteins were extracted from collected leaves. The phosphoproteins were enriched by the phosphoprotein enrichment kit (Pierce^TM^), tryptic digested, and analyzed by LC-MS. Decyder MS software was used for the quantification of the MS/MS intensity in each sample, and peptide sequences were searched against the NCBI protein database using Mascot software. A total of 4721 phosphoproteins were detected in LPT123 and KDML105 rice cultivars among the four comparison groups (CON, CTS, CON + PEG, and CTS + PEG). In the LPT123 cultivar, 832 DEPs were common to all groups. Before osmotic stress, 238 (I) or 258 (II) DEPs were independently observed in the CON and CTS groups. After osmotic stress, 309 (III) or 247 (IV) DEPs were independently expressed in the CON + PEG or CTS + PEG treatments. Of these, 141 DEPS were found in the CON and CTS treatments, whereas 165 DEPs were detected in the CON + PEG and CTS + PEG treatment groups (Figure 2a and Table S1). Moreover, in the KDML105 cultivar, 790 DEPs were identified in all treatment groups. A total of 264 (V), 254 (VI), 256 (VII), or 266 (VIII) DEPs were uniquely detected in the CON, CTS, CON + PEG, or CTS + PEG treatment groups, respectively. The CON and CTS treatments contained 204 common DEPs, whereas 158 DEPs were identified after osmotic stress treatment in the CON + PEG and CTS + PEG groups (Figure 2b and Table S2).

**Figure 3:**
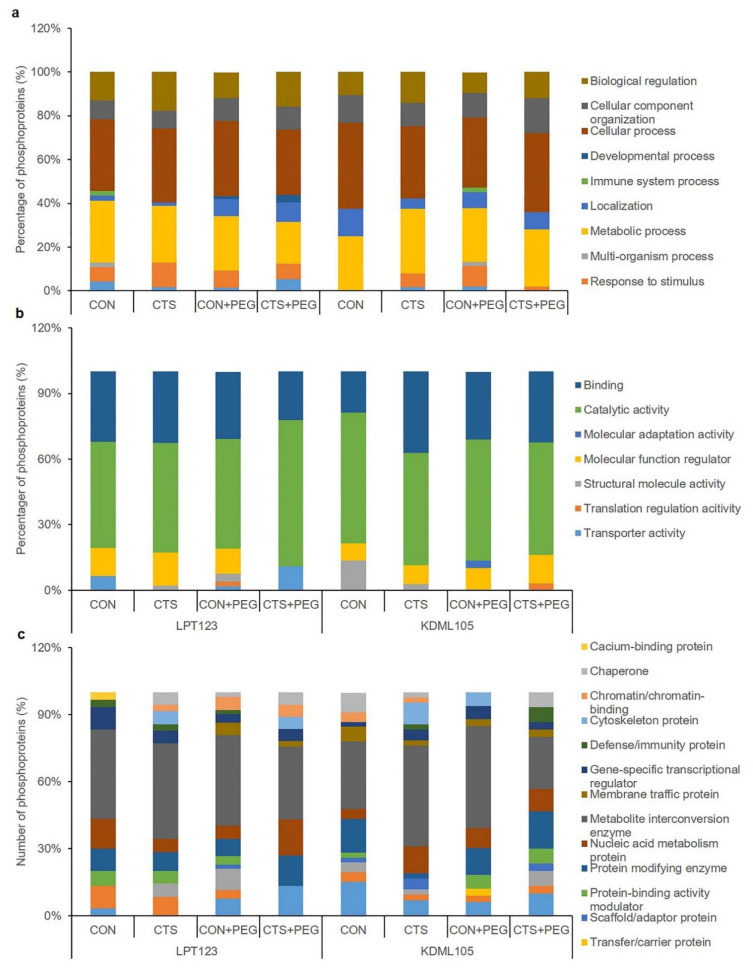
Gene ontology (GO) analysis provided three categories, including biological processes (**a**) molecular function; (**b**) and cellular components (**c**) of DEPs of LPT123 and KDML105 rice cultivars.

The output of unique DEPs in LPT123 (I–IV) and KDML105 (V–VIII) from the Venn diagram were annotated using the PANTHER web-based application. GO annotation yielded three categories: biological process, molecular function, and protein class (Figure 3). The independent proteins in the rice leaves of the different treatment groups, based on the relative percentage in the biological processes, were as follows: biological regulation, cellular component organization, cellular process, developmental process, immune system process, localization, metabolic process, multi-organism process, response to stimulus, and signaling. Cellular and metabolic processes contained the majority of proteins for all treatment groups of both rice cultivars, whereas the highest percentage (5%) of protein involved in signaling was identified in the CTS + PEG treatment of LPT123 (Figure 3a). The unique DEPs assigned to the molecular function category were further annotated as binding, catalytic activity, molecular adaptation activity, molecular function regulator, structure molecule activity, translation regulation activity, and transporter activity. Of these annotated proteins, catalytic activity was the predominant functional classification of the LPT123 and KDML105 cultivars, which showed the highest percentage of proteins (48%–67%) in the CTS + PEG treatment in LPT123. In addition, transporter activity was only observed in the LPT123 cultivar and showed the highest percentage of proteins (11%) in the CTS + PEG-treated plants (Figure 3b). Moreover, the identified proteins assigned to the protein class were annotated as follows: calcium-binding protein, chaperone, chromatin/chromatin-binding, cytoskeleton protein, defense/immunity protein, gene-specific transcriptional regulator, membrane traffic protein, metabolite interconversion enzyme, nucleic acid metabolism protein, protein-modifying enzyme, protein-binding activity modulator, scaffold/adaptor protein, transfer/carrier protein, translational protein, transmembrane signal receptor, and transporter categories. The most common protein class was metabolite interconversion enzyme (23%–46%) (Figure 3c).

### 2.3. Clustering Analysis and Identification of CTS-Responsive Proteins

**Figure 4:**
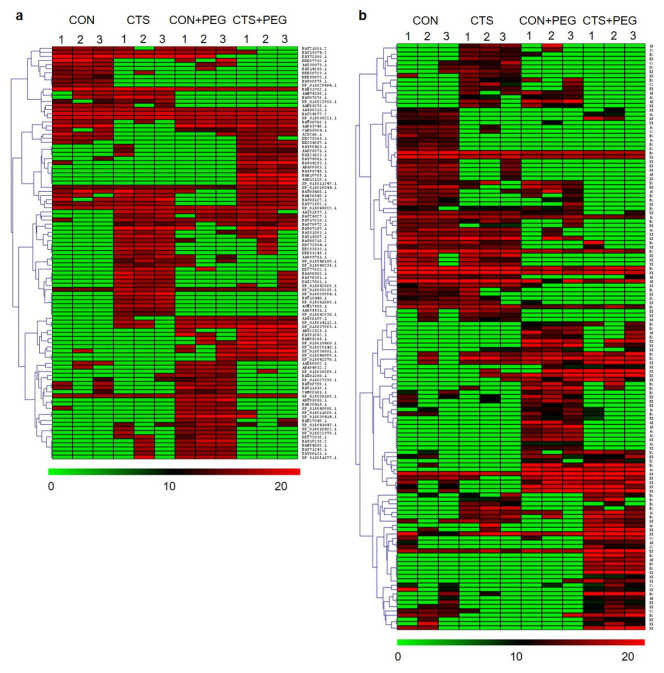

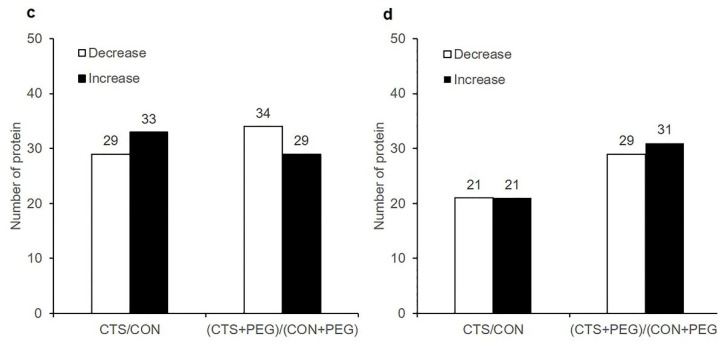
Heat map of significant DEPs of LPT123 (**a**) KDML105; (**b**) cultivars in the four treatment groups. The heat map was created using a MultiExperiment Viewer. Each row in the hierarchical clustering analysis represents an individual protein. The color scale (green to red) indicates low to high protein expression, and the numbers above the column refer to the number of replications. This shows the number of significant DEPs with and without CTS application in unstressed (CON and CTS) and stressed plants (CON + PEG and CTS + PEG) of LPT123; (**c**) and KDML105; (**d**) rice leaves.

To examine the CTS-response proteins during osmotic and non-osmotic stress conditions, we conducted a hierarchical clustering analysis of the DEPs from the four different groups (CON, CTS, CON + PEG, and CTS + PEG) by comparison with and without CTS-treated plants at the initiation of osmotic stress (CTS and CON) and after osmotic stress (CTS + PEG and CON + PEG) in the LPT123 (Figure 4a) and KDML105 (Figure 4b). A total of 125 and 102 significant DEPs were detected in LPT123 and KDML105, respectively. In the LPT123 rice leaves, 62 DEPs (29 decreased and 33 increased) were induced by CTS treatment at the initiation of osmotic stress (day 0). In comparison, 63 DEPs (34 decreased and 29 increased) were induced by CTS after 3 days of osmotic stress (Figure 4c). Additionally, 42 DEPs (21 decreased and 21 increased) were detected under unstressed conditions, whereas 60 DEPs (29 decreased and 31 increased) were identified after CTS treatment and osmotic stress in KDML105 rice leaves (Figure 4d). Of these, the levels of 43 DEPs increased in both rice cultivars with known annotated functions, whereas the remaining were hypothetical or unknown proteins. Based on the biological process, the DEPs were involved in signal transduction, defense response, transport, replication, transcription, translation, protein folding/degradation, and metabolic processes. In addition, the 21 DEPs were identified as exhibiting significant changes in response to CTS induction without being subjected to PEG treatment, of which 14 and 7 DEPs were in the LPT123 and KDML105 cultivars, respectively (Table 1). Finally, 22 DEPs were found in response to CTS induction with osmotic stress treatment, of which 10 and 12 were present in the LPT123 and KDML105 cultivars, respectively (Table 2).

### 2.4. Identification of Phosphorylated Proteins and Phosphorylation Motifs Induced by CTS Treatment

Among the CTS-responsive phosphoproteins, significant changes were detected in phosphorylation sites under non-osmotic stressed and osmotic stressed conditions in the two rice cultivars. Under non-osmotic stress, seven phosphorylated proteins, including adenylyltransferase and sulfurtransferase MOCS3 (BAS81451.1), NBS-LRR disease resistance protein (ACM17588.1), DEAD-box ATP-dependent RNA helicase 18 (XP_015620894.1), La-related protein 1A isoform X1 (XP_015621184.1), septum site-determining protein mind homolog (XP_015642930.1), KAT8 regulatory NSL complex subunit 2 (BAT16440.1), and peroxygenase 4 (XP_015641088.1), were identified in LPT123. Chalcone synthase (BAD53112.1) was also observed in the CTS-treated KDML105 cultivar (Table 1). Under osmotic stress, four phosphoproteins were identified in CTS-treated LPT123, including beta-amylase 1 (AFI71858.1), chaperone protein dnaJ 49 (BAS93628.1), ABC transporter C family member 4 (XP_015611347.1), and cytochrome b561 (XP_015612553.1). In addition, eight phosphoproteins were induced by CTS treatment in KDML105 plants, which included receptor-like serine/threonine-protein kinase (XP_015617069.1), ubiquitin carboxyl-terminal hydrolase 15 (XP_015627057.1), U-box domain-containing protein 45 (XP_015613842.1), Na/H antiporter (AAQ74383.1), auxin transport protein BIG (B9G2A8.1), B3 domain-containing protein Os02g0598200 (XP_015624189.1), lipase family protein (ABA95184.1), and branched-chain amino acid aminotransferase (ABF96062.1) (Table 2). Based on the identified phosphorylation sites, an analysis of Ser, Thr, and Tyr residues revealed that peptides which had specific phosphorylation characteristics in response to CTS induction showed significant changes in phosphorylation sites. Remarkably, four phosphoproteins that were induced by CTS during osmotic stress contained three different Ser and Thr sites. The phosphorylation of SGDTSSR (cytochrome b561) at Thr4, Ser5, and Ser6 sites was identified in LPT123, whereas RNSPNSIDSK (branched-chain amino acid aminotransferase), SLHSPLLTR (Na/H antiporter), and KLGSSILSSR (auxin transport protein BIG) were identified in KDML105 plants (Table 2). These results indicate that CTS pretreatment under osmotic stress generates phosphoproteins with multiple phosphorylated sites in a single protein.

## 3. Discussion

### 3.1. CTS Affected Growth Enhancement and Photosynthetic Pigments in the Osmotic Stress Condition

Several studies on drought stress responses have been conducted using rice seedlings transferred into nutrient solutions containing the agent PEG6000 [18] to create osmotic stress. Drought stress reportedly inhibits plant growth, especially in sensitive rice cultivars. However, applying CTS stimulates plant growth under osmotic stress in several plants. Similarly, in the current study, exogenous CTS treatment during osmotic stress resulted in significant osmotic resistance and improved shoot growth (SFW and SDW) in KDML105 (Figure 1a,b). Drought stress impairs photosynthetic ability, thus reducing chlorophyll synthesis. This may be due to the destruction of chlorophyll pigment complexes. In this study, CTS-treated LPT123 rice seedlings could elicit photosynthetic pigment contents (Chl *a*, Chl *b*, and carotenoids) under osmotic stress (Figure 1c–e). CTS pretreatment increased photosynthetic pigments in thyme [8], annual ryegrass [14], and creeping bentgrass [20]. The action of CTS could induce the photosynthetic pigment contents and increase level of several proteins in chloroplasts, suggesting that the chloroplast is a target organelle [15] and CTS affected chloroplast enlargement in *Dendrobium* orchids [21]. CTS activity was previously reported to vary depending on the plant species and genotypes. As LPT123 and KDML105 were proposed to be drought-sensitive rice cultivars and differed in CTS response, these provide an excellent model for identifying the required mechanism for CTS-induced resistance to osmotic stress at the posttranslational level.

### 3.2. Quantitative Phosphoproteomics Analysis as a Powerful Tool for Analysis of Leaf Phosphoproteins

Phosphoproteomics has been proposed as an effective tool for studying plant response to drought stress. Phosphorylation of proteins is central to several metabolic, hormonal, developmental, and stress responses, and is extensively employed in signal transduction, frequently involving cascades of protein kinases and phosphatases [22]. Protein phosphorylation seems to be regulated by the coordinated actions of protein kinases and phosphatases, which account for about one-third of all proteins in eukaryote cells [23]. Detecting changes in protein phosphorylation can be a difficult task because of the transient labile state of the phosphate group. Furthermore, low phosphoprotein abundance and poorly developed phospho-specific antibodies also contribute to difficulties in phosphoprotein detection. As a result, phosphoproteome analysis necessitates highly sensitive and specific methods. Currently, the majority of phosphoproteomic studies are performed by mass spectrometric approaches combined with phospho-specific enrichment methods [16]. Using quantitative proteomics approaches, these variations at the protein level can be detected and measured, providing valuable information about the understanding of molecular mechanisms. In the present study, we conducted a gel-free-based quantitative phosphoproteomics analysis of the chitosan response to short-term osmotic stress in rice leaves. A large number of phosphoproteins were identified. These chitosan-responsive phosphoproteins and the related signaling and metabolic pathways might play important roles in chitosan signaling and response to osmotic (drought) stress in rice leaves.

### 3.3. Protein Kinases Associated with Signal Transduction Induced by CTS and the Stress Response

Protein phosphorylation plays an important role in complex signaling networks and regulates a wide range of cellular processes, such as transmembrane signaling, intracellular amplification of signals, hormone sensing, and environmental stress response [24]. In the present study, three kinases were significantly increased in response to CTS under osmotic stress. For example, histidine-kinase-like protein (AAK13126.1) and protein kinase domain (AAX95871.1) were significantly attenuated in CTS-treated LPT123 leaves. Receptor-like serine/threonine-protein kinase (XP_015617069.1) was phosphorylated at a Thr residue and was increased in the KDML105 cultivar (Table 2). Most histidine protein kinases are transmembrane protein receptors for extracellular signals [25]. In *Arabidopsis*, histidine kinases have different functions, including ethylene signaling, osmosensing, and cytokine signaling, and their respective genes are involved in drought response [26]. Previous studies have indicated that receptor-like serine/threonine-protein kinases play a key role in regulating plant response to drought stress in wheat leaves [1]. A phosphoproteome analysis revealed a significant change in serine threonine-protein kinase wnk-4-like during drought stress in maize leaves [27]. Additionally, at the initiation of PEG treatment, receptor-like protein kinase (BAT07207.1) increased in CTS-treated LPT123 cultivar (Table 1). It has been reported that CTS induces a receptor-like kinase gene, the MAP kinase signaling pathway, and lysine motif receptor-like kinase, chitin elicitor receptor kinase 1 (CERK1), which binds to chitin and CTS [28]. In contrast, some studies have indicated that CTS signaling does not activate the CERK1-independent pathway in *Arabidopsis* seedlings [29]. Our findings suggest that CTS pretreatment before exposure to osmotic stress in rice seedlings induces various protein kinases involved in signaling and may be involved in the drought response, particularly in the LPT123 cultivar. It is important to note that the protein kinases identified in this study should be further experimentally validated.

### 3.4. Phosphoproteins Involved in the Defense Response

Plants respond actively to stress by producing stress metabolites. Stress can result from injuries caused by insects and microbes or by mechanical wounds, which can induce many distinct biochemical changes. These include the production of protective compounds either at the site of injury or systemically in distant unwounded tissues. In plants, phenylalanine is derived from the precursor, chorismite, which is involved in the flavonoid and phenylpropanoid pathways. Flavonoids play an important role in plant defense, and chalcone synthase, as the gatekeeper of flavonoid biosynthesis, plays a central role in regulating this pathway [30]. In the present study, the chalcone synthase (BAD53112.1) protein was phosphorylated and increased in CTS-treated KDML105 during non-osmotic stress. This phosphoprotein is a key enzyme of the flavonoid/isoflavonoid biosynthesis pathway. Additionally, chalcone synthase gene expression is influenced by stress and environmental factors such as UV, wounding, or pathogen attack. Similarly, we found that phenylalanine ammonia-lyase (BAF29919.2) was expressed in CTS-treated KDML105 rice. Previous reports have indicated that CTS induces resistance against *Blumeria graminis* f. sp. *hordei* in barley plants via oxidative burst induction and phenolic compound deposition [31]. In addition, this significantly improved phenol accumulation and flavonoid metabolism in white clover [13].

CTS can elicit plant defense responses to wounds and pathogen infections. It may cause tomato (*Solanum lycopersicum* L.) plants to generate a proteinase inhibitor and pea (*Pisum sativum* L.) pods to release phytoalexin (pisatin). CTS may also influence the expression of genes that respond to biotic stress, such as chitinase and glucanase [32]. In the present study, the NBS-LRR disease resistance protein (ACM17588.1) and disease resistance protein, RPM1 (BAF05127.1), were increased in the CTS-treated LPT123 cultivar under non-osmotic and osmotic stress conditions, respectively. NBS-LRR proteins act through a network of signaling pathways to promote a series of plant defense responses, such as activation of an oxidative burst, calcium and ion fluxes, mitogen-associated protein kinase signaling, induction of pathogenesis-related genes, and the hypersensitive response [33]. Moreover, the RPM1 protein functions by regulating a gene-for-gene process in which pathogen-encoded virulence gene products are recognized explicitly by plant disease resistance gene products, either directly or indirectly. It is involved in the hypersensitive response, which suggests a negative feedback loop that regulates the level of cell death and overall resistance at the infection site [34]. These results suggest that CTS could increase the expression of several phosphoproteins involved in the defense response, which is comparable to the pathogen/wound response.

### 3.5. Phosphoproteins Involved in Transmembrane Transport

Transporter proteins are important for maintaining turgor pressure and regulating water potential, both of which are essential for plant growth and survival under abiotic stress. In the present study, CTS increased the expression of ABC transporter C family member 4 (XP_015611347.1), which was increased in LPT123 during osmotic stress (Table 2). The ABC transporters have an essential role in plant growth, development, response to abiotic stress, the interaction of the plant with its environment, and in the transport of auxin and abscisic acid [35]. The results of transcriptional profiling of *Arabidopsis* seedlings indicated that CTS induced the ABC transporter family (*PDR12*)*,* which is involved in the jasmonic response [29]. CTS treatment may alleviate drought damage relative to an increase in abscisic acid content [7]. Srivastava et al. [36] proposed that the effects of CTS are similar to that of abscisic acid or jasmonic acid on guard cells, indicating the coordination of their signal transduction pathways, which results in stomatal closure. Another report indicated that CTS also causes auxin accumulation by blocking *PIN1* gene expression, which is involved in auxin transport and results in decreased primary root length and secondary root emergence [37]. Interestingly, in the present study, the auxin transport protein BIG (B9G2A8.1) was identified and increased in KDML105 rice leaves under osmotic stress. This phosphoprotein may enhance plant growth and may participate in hormone signaling and transport in response to CTS induction. The B3 domain-containing protein, Os02g0598200 (XP_015624189.1), was identified as a transcription factor (TF) that triggers various signaling pathways. Previous studies also indicated that CTS induced the expression of transcription factors, which participate in TF-mediated embryo axis formation and vascular development, similar to TF auxin response factor 1 [38]. Auxin plays an important role in meristematic cell differentiation in the shoot apical meristem and is an important signal of abiotic stress [39]. In addition, it has been reported that CTS-pretreated plants exhibit significantly higher Na^+^ content in roots and lower Na^+^ accumulation in leaves compared with untreated plants in response to salt stress and enhanced salt overly sensitive pathways. The expression of *AsHKT1* and the genes *AsNHX4*, *AsNHX5,* and *AsNHX6* encode Na^+^/H^+^ exchangers during salt stress [40]. In this study, the Na/H antiporter (AAQ74383.1) responded to CTS induction in the KDML105 cultivar, suggesting that CTS induced this phosphoprotein to regulate the osmotic balance of rice seedlings during osmotic stress.

### 3.6. Phosphoproteins Involved in Transcription

To generate translatable mRNAs, pre-mRNA molecules must be appropriately processed, spliced, and delivered to the cytoplasm. In the present study, many phosphoproteins involved in the posttranscriptional regulation of RNA were identified, which highlights their roles in response to CTS during non-PEG treatment. In LPT123 plants, La-related protein 1A isoform X1 (XP_015621184.1) is implicated in the stability, localization, and translational efficiency of mRNAs required for cell development, migration, division, and general translation [41]. In addition, DEAD-box ATP-dependent RNA helicase 18 (XP_015620894.1) was identified in rice leaves. The chloroplast-localized *Arabidopsis* AtRH3 has an important role in intron splicing, ribosome biogenesis, and seedling growth [42]. DEAD-box RNA helicases are targeted to chloroplasts. In addition, septum site-determining protein minD homolog (XP_015642930.1) was proposed as a membrane ATPase. The overexpression of the *Arabidopsis* MinD homolog, *AtMinD1*, is associated with chloroplast division and alters chloroplast size and number in transgenic tobacco plants [43]. Consistent with this finding, the chloroplast is believed to be the primary organelle for CTS action. The expression of the chloroplast gene, *ycf1*, was detected after a 48-hour treatment with CTS in *Dendrobium* “Eiskul,” followed by chloroplast enlargement after 68 weeks [21]. A subsequent proteomics analysis for CTS-responsive proteins in rice leaves revealed that CTS induced significant changes in the expression of 352 proteins. A network analysis revealed that many coexpressed proteins were localized in the chloroplasts [15].

### 3.7. Phosphoproteins Involved in Protein Folding and Degradation

Heat-shock proteins (Hsps)/chaperones are responsible for protein folding, assembly, translocation, and degradation during many normal cellular processes. They stabilize proteins and membranes and assist in protein refolding under stress conditions [44]. In the present study, two chaperone proteins, DnaJ 49 (BAS93628.1) and T-complex protein (BAS95271.1), were increased in CTS-treated LPT123 under osmotic stress. The DnaJ protein constitutes a DnaJ/Hsp40 family member and is an important regulator of diverse cellular functions. The putative DnaJ protein ortholog from *Nicotiana tabacum* may be involved in drought stress response, and its overexpression enhances drought tolerance, possibly by regulating stress-responsive gene expression. The increased expression of *RD20*, *RD22,* and *AREB2* in NtDnaJ1 transgenic plants indicates that it may participate in the ABA-dependent signaling pathway during drought stress [45].

The ubiquitin/26S proteasome system is another mechanism for protein degradation. During abiotic stress, it eliminates misfolded or damaged proteins and manages the quantity of specific regulatory proteins [46]. The ubiquitin carboxyl (C)-terminal hydrolase 15 protein is linked to the proteolysis of signaling pathways involved in environmental adaptation in higher plants [47]. It can hydrolyze a variety of ubiquitin linkages either before or after proteolysis and plays a role in recycling ubiquitin and reversing ubiquitin conjugation during signal transduction [48]. The U-box domain-containing protein acts as a single peptide E3 ligase and may be involved in transcription-dependent resistance to drought stress in plant cells [49]. Phosphoprotein analysis of maize leaves revealed that the E3 ubiquitin ligases, rglg2-like isoform x1 and upl4-like, are expressed during drought stress [27]. Our data showed that E3 ubiquitin–protein ligase (BAS70391.1) was expressed in CTS-treated LPT123 before exposure to osmotic stress, whereas ubiquitin carboxyl-terminal hydrolase 15 (XP 015627057.1) and U-box domain-containing protein 45 (XP 015613842.1) were detected in CTS-treated KDML105 during osmotic stress (Table 1 and Table 2). Alterations in phosphoproteins caused by exogenous CTS may regulate protein folding and degradation associated with signaling and the stress response.

### 3.8. Phosphoproteins Relative to Plant Metabolism

Carbohydrates are abundant and significant during plant stress defense, and they function as compatible solutes, energy sources, and signaling molecules [13]. In the present study, we showed that β-amylase 1 (BAM1; AFI71858.1) was increased in CTS-treated LPT123 plants during osmotic stress. Zanella et al. [50] proposed that BAM1 degrades transitory starch to sustain proline biosynthesis during drought stress. Additionally, *bam1* mutants show reduced proline accumulation and suffer from stronger lipid peroxidation compared with the wildtype.

Amino acids are well-known stimulants that positively impact plant growth and yield while also functioning as suitable osmolytes to help plants recover from abiotic stress [51]. A previous study found that CTS treatment enhanced the production of metabolites and amino acids, such as proline, asparagine, valine, serine, leucine, threonine, isoleucine, and phenylalanine in white clover (*T. repens* L.) [13]. Our results support the previous finding that branched-chain amino acid aminotransferase (BCAT; ABF96062.1) is increased in CTS-treated KDML105 plants during osmotic stress. This phosphoprotein is associated with amino acid metabolism by participating in valine, leucine, and isoleucine formation, the small group of branched-chain amino acids in *Arabidopsis*, which are classified by their small branched hydrocarbon residues [52]. CTS contributes to carbohydrate and amino acid metabolism, which may be particularly strong under osmotic stress conditions.

CTS affects the membrane permeability in cell suspensions of soybean (*G.* *max* (L.) Merr.) and common bean (*P. vulgaris* L.) [53]. The incubation of excised grapevine leaves in 75–150 mg/L crab shell CTS resulted in the induction of lipoxygenase (LOX) activity [54], causing peroxidation, which may play a role in octadecanoid defense signaling pathway, leading to systemic acquired resistance [46]. Consistent with this finding, CTS induced increased levels (bursts) of 12-oxo-phytodieonic acid, an octadecanoid signaling component in rice seedlings [55]. Similarly, in the present study, peroxygenase 4 (XP_015641088.1) was identified in the CTS-treated LPT123 rice cultivar during non-osmotic stress. The peroxygenase pathway also constitutes one branch of the so-called “LOX pathway” as the first step. LOX catalyzes the oxygenation of unsaturated fatty acids (C18:2, C18:3, or C16:3) and yields the corresponding fatty acid hydroperoxides [56]. Peroxygenase is a hydroperoxide-dependent oxygenase that catalyzes the transfer of one oxygen atom from a hydroperoxide to an oxidized substrate [57]. It is associated with bilayer membranes and/or lipid droplets, with some isoforms binding to both types of lipid structures [58]. In addition, lipase family protein (BA95184.1) was found in CTS-treated KDML105 rice during osmotic stress. GDSL-type esterase/lipase proteins are various hydrolytic enzymes (lipolytic enzymes) with broad substrate specificity that can hydrolyze many substrates, such as thioesters aryl esters, phospholipids, and amino acids. GDSL family members are essential for the regulation of plant growth and development, secondary metabolism, plant immunity, and abiotic stress [59]. Our results suggest that these phosphoproteins induced by the CTS induction may be involved in the octadecanoid signaling pathway at the cellular level in the KDML105.

### 3.9. Predicted Phosphorylation Sites of the Peptides

Site-specific protein phosphorylation is one of the most important post-translational modifications (PTMs). It can rapidly modulate a protein’s function by changing its activity, subcellular localization, interactions, or stability. It is a highly dynamic modification that regulates all cellular signaling networks [60]. Peptide phosphorylation is a peptide containing phosphate group for a peptide containing Ser, Thr, or Tyr. Phosphorylation on these amino acids is an essential modulator of post-translational modification of protein function and is associated with many proteins that have a regulatory function in cells. Protein phosphorylation can occur in several amino acids. Phosphorylation on Ser is the most common, followed by Thr. Tyr phosphorylation is relatively rare but is at the origin of protein phosphorylation signaling pathways in most eukaryotes. In the present study, several peptides were phosphorylated at the Ser site, such as cytochrome b561 (XP_015611347.1), Na/H antiporter (AAQ74383.1), and auxin transporter protein BIG (B9G2A8.1) identified in LPT123 and KDML105 under osmotic stress. It was reported that the phosphorylated serine-rich domain, as a nuclear localization signal (NLS), can allow dehydrins (DHNs) to enter the nucleus and act as chaperones and cytoskeleton regulation [61]. Based on the results above, we boldly speculate that their significant phosphorylation may form a signal peptide to navigate to the nucleus. They are likely to be some candidates for CTS-induced phosphoproteins for drought resistance study.

## 4. Materials and Methods

### 4.1. Plant Material, Growth Conditions, CTS, and Osmotic Stress Treatments

Seeds from two rice (*O.* *sativa* L.) cultivars, LPT123 and KDML105, were provided by the Agriculture Department Ministry of Agriculture and Cooperation, Thailand. Rice seeds were first soaked for 48 h in a 40 mg/L solution of 80% deacetylated oligomeric CTS (Olizac Technologies, Pathum Thani, Thailand) or distilled water. The seeds were germinated in plastic containers (36 cm in length, 30 cm in width, and 9.5 cm deep) filled with sterilized sand, for two weeks. Plant seedlings were transferred to containers for culturing and filled with WP nutrient solution [62]. CTS at the same concentration, containing 0.01% (*v/v*) Triton X-100, was applied to the 2- and 4-week-old seedlings by spraying until they were fully soaked. The control treatment was performed by spraying with distilled water mixed with 0.01% (*v/v*) Triton X-100 during the same period before exposure to osmotic stress. All rice seedlings were grown in a greenhouse under 400 ± 50 μmol/m^2^/s photosynthetic photon flux density and a temperature shift at 32 °C ± 2 °C/28 °C ± 2 °C during day/night intervals. Four replicates for each treatment were arranged in a completely randomized design. CTS and non-CTS-treated plants were grown in WP nutrient solution containing 10% (*w/v*) polyethylene glycol 6000 (PEG6000) (as a surrogate model of osmotic) stress two days after the last CTS or without CTS treatment. Leaf samples (6 plants/replicate) were collected during 7 days of osmotic stress from four independent replicates to determine shoot fresh weight (SFW), shoot dry weight (SDW) and photosynthetic pigments.

**Figure 5:**
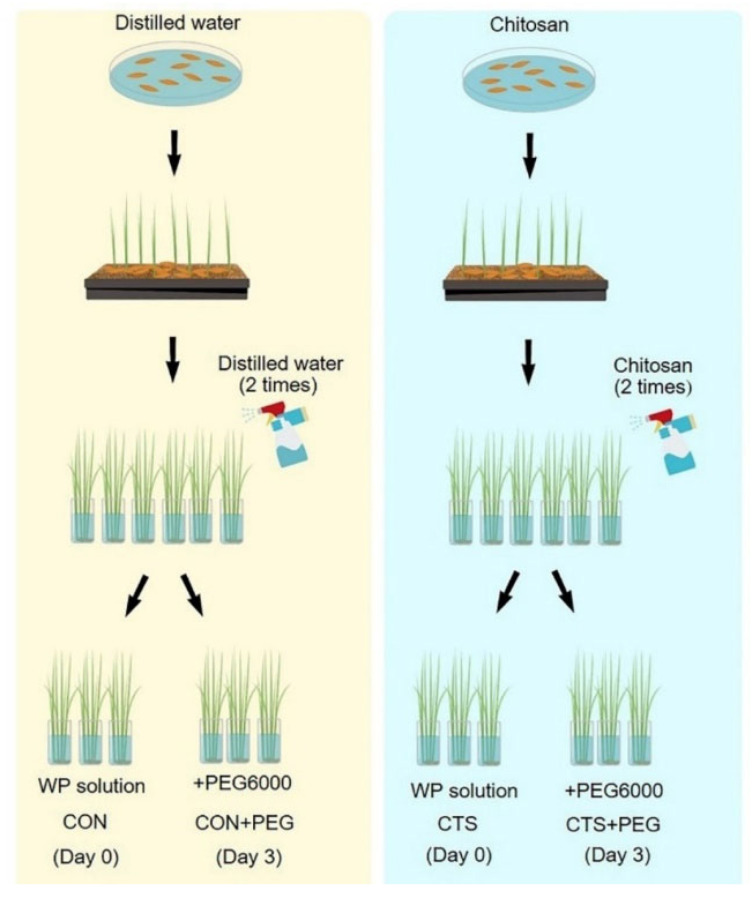
Scheme of the methodology for four different treatments: (1) CON, seedlings treated with distilled water; (2) CTS, seedlings treated with CTS; (3) CON + PEG, seedlings pretreated with distilled water followed by PEG6000; and (4) CTS + PEG, seedlings pretreated with CTS followed by PEG6000. Rice seedlings were harvested for phosphoproteome analysis on day 0 (CON and CTS) and day 3 (CON + PEG and CTS + PEG) after osmotic stress treatment.

For the proteomics analysis, three biological replicates were established in a completely randomized design. Leaf tissues from twenty rice seedlings for each biological replicate were harvested on day 0 and day 3 after osmotic stress treatment. The four different treatments were as follows: (1) CON, seedlings treated with distilled water; (2) CTS, seedlings treated with CTS; (3) CON + PEG, seedlings pretreated with distilled water followed by PEG6000; and (4) CTS + PEG, seedlings pretreated with CTS followed by PEG6000. (Figure 5). 

### 4.2. Photosynthetic Pigments Determination

The Chl *a*, Chl *b*, and carotenoid contents were measured following the methods of Pongprayoon et al. [19]. Briefly, 200 mg of fresh leaves were homogenized with 5 mL of 80% (*v/v*) acetone, then wrapped in aluminum foil and placed in a refrigerator for 48 h. The Chl *a* and Chl *b* were quantified at wavelengths of 662 and 644 nm, whereas carotenoids were determined at 470 nm on a microplate spectrophotometer (Multiscan GO; Thermo Fisher Scientific, Waltham, MA, USA). The 80% (*v/v*) acetone solution was used as a blank control. 

### 4.3. Total Protein Extraction

The extraction procedure for phosphoproteomic analysis was conducted in accordance with the method described by Pongprayoon et al. [19]. Approximately 0.5 g of fresh leaf tissue was ground into a fine powder in liquid nitrogen and resuspended in 900 mL of 0.5% (*w/v*) sodium dodecyl sulfate (SDS). The mixture was thoroughly vortexed for 30 min and centrifuged at 11,290× *g* at 4 °C for 15 min. The precipitated proteins were washed twice with an equal volume of cold acetone. The protein pellet was resuspended in 0.5% (*w/v*) SDS and the protein concentration was determined by the Lowry method [63] using bovine serum albumin as a standard.

### 4.4. Phosphoprotein Enrichment and Digestion

Phosphoproteome analysis was performed according to the phosphoproteome workflow procedure described by Nakagami et al. [64]. One hundred micrograms of protein were used for phosphoprotein enrichment using the Pierce Phosphoprotein Enrichment Kit according to the manufacturer’s instructions and desalted on HiTrap Desalting Columns (Merck KGaA, Darmstadt, Germany). The phosphoproteins were reduced with 10 mM dithiothreitol (DTT), alkylated with 30 mM iodoacetamide (IAA) in 10 mM ammonium bicarbonate, and digested with sequencing-grade trypsin (Promega Corporation, Fitchburg, WI, USA) for 16 h at 37 °C. Trypsin-digested peptides were then concentrated using a SpeedVac Vacuum Concentrator (Thermo Fisher Scientific, Vantaa, Finland) and dissolved in 0.1% formic acid (FA) for MS analysis.

### 4.5. Liquid Chromatography–Mass Spectrophotometry (LC-MS/MS)

Phosphopeptide samples were subjected to liquid chromatography (LC) using an Ultimate 3000 LC System (Dionex Corporation, Sunnyvale, CA, USA) coupled with an ESI ion trap mass spectrometer (HCT Ultra PTM Discovery System; Bruker Daltonik GmbH, Bremen, Germany) and equipped with a monolithic nanocolumn (100-µm i.d. × 5 cm; Thermo Fisher Scientific Vantaa, Finland) at an electrospray flow rate of 20 µL/min and a mobile phase flow rate of 0.3 µL/min. The mobile phase consisted of a nonlinear gradient of solvent A [0.1% (*v/v*) formic acid in H_2_O] and solvent B [20% (*v/v*) H_2_O, 80% (*v/v*) acetonitrile, 0.1% (*v/v*) formic acid] changing from 9:1 (*v/v*) A:B to 3:7 (*v/v*) A:B from 0 to 13 min, then 1:9 (*v/v*) A:B from 13 to 15 min, and 9:1 (*v/v*) A:B from 15 to 20 min. Electrospray ionization was performed at 1.6 kV using CaptiveSpray. Nitrogen was used as a drying gas (flow rate about 50 L/h). Collision-induced dissociation product ion MS were obtained using nitrogen gas as the collision gas. MS and MS/MS spectra were obtained in the positive-ion mode at 2 Hz over the range (m/z) 150–2200. The collision energy was adjusted to 10 eV as a function of the m/z value.

### 4.6. Data Analysis and Bioinformatics

DeCyder MS 2.0 analysis software (GE Health-care, Chicago, IL, USA) was used to measure the relative protein abundance based on MS peptide signal intensities of individual LC-MS analyzed data. An average abundance ratio of more than two-fold was designated an overexpressed protein with a significant standard *t*-test and a one-way ANOVA (*p* < 0.05). All MS/MS spectra from the DeCyder MS were analyzed by applying the global variable mode of carbamidomethyl, variable mode of oxidation (M), phospho (ST), and phospho (Y), peptide charge states (1+, 2+, and 3+), and an m/z tolerance 0.1 µ. The spectra were searched against the NCBI protein database (https://www.ncbi.nlm.nih.gov/; 23 March 2019) using the *O. sativa* L. proteome to identify matching peptides using the Mascot software search engine (Matrix Science, London, UK). The identified proteins were filtered with a one-way ANOVA *p* < 0.05. In this experiment, 200 fg of BSA was used as an internal standard to normalize protein intensities for each data set. Functional classification and annotation of the proteins were done using Gene Ontology (GO), the Protein ANalysis THrough Evolutionary Relationships (PANTHER) classification system (http://www.pantherdb.org/; 18 December 2020) [65], and UniProt (https://www.uniprot.org/; 19 December 2020). The levels of significantly expressed proteins from the hierarchical clustering analysis were determined using MultiExperiment Viewer (MeV) software (Version 4.8.1) [66]. The identified phosphoproteins were used to predict phosphorylation using their peptide sequences with the NetPhos 3.1 server (https://services.healthtech.dtu.dk/service.php?NetPhos-3.1; 17 April 2022). The program identifies specific residues, such as p-threonine (Thr), p-serine (Ser), and p-tyrosine (Tyr) phosphorylation sites [62]. The prediction score is a value in the range of 0.000–1.000, and scores above 0.500 indicate positive predictions.

### 4.7. Statistical Analysis

The data of SFW, SDW and photosynthetic pigments were performed using analysis of variance (ANOVA) and the mean comparison was performed with Duncan’s multiple range test (DMRT), establishing statistical significance at *p*-values of *p* < 0.05. The bars in all figures represent the standard deviation of the mean. 

The hierarchical clustering analysis was determined using MEV software by the maximum protein intensity of the three replicates and was subjected to a *t-test* to determine statistical significance. A *P*-value < 0.05 was considered statistically significant.

## 5. Conclusions

This present study revealed changes in the phosphoproteome of rice seedlings induced by CTS pretreatment followed by exposure to osmotic stress. Phosphoproteome analysis identified a significant number of DEPs and phosphorylation sites that are involved in signaling, transport, protein folding, degradation, and metabolism in response to CTS induction and osmotic stress. The increase in CTS-induced phosphoproteins after osmotic stress may enhance the drought resistance in LPT123 and KDML105 cultivars. In addition, some differentially expressed phosphoproteins found in LPT123 or KDML105 may reflect cultivar differences and contribute to CTS-induced resistance to the osmotic stress of each cultivar. Our results extend the current knowledge regarding plant response to CTS under osmotic stress through the application of the phosphoproteomic approach.

## Figures and Tables

**Table 1:** The increased DEPs in both rice cultivars in response to CTS induction without osmotic stress treatment.

Cultivar	Accession Number	Protein Name	Peptide	Peptide Sequence and Predicted Phosphorylation Site (*)	Score	Intensity		Functional Category
						CON	CTS	
**LPT123**	BAS81451.1	Adenylyltransferase and sulfurtransferase	AINSSIK	AINS*SIK	0.598	0.0000	16.7085	Signal transduction
	BAS81893.1	Os03g0108200	FAMASRPR	FAMASRPR		0.0000	16.9662	Signal transduction
	BAT07207.1	Receptor-like protein kinase	VLLELVHGRK	VLLELVHGRK		0.0000	22.9446	Signal transduction
	ACM17588.1	NBS-LRR disease resistance protein	NLRYLNLAR	NLRY*LNLAR	0.879	0.0000	17.6093	Defense response
	AAX95751.1	WAT1-related protein	MTSSSLK	MTSSSLK		0.0000	18.0284	Transport
	AAX95531.1	Swi1	VRYIGR	VRYIGR		0.0000	15.5903	Transcription
	BAT16440.1	KAT8 regulatory NSL complex	GLLQLHSLYR	GLLQLHS*LYR	0.537	0.0000	17.5308	Transcription
	XP_015620894.1	DEAD-box ATP-dependent RNA helicase	QRQAVQTK	QRQAVQT*K	0.693	0.0000	21.8363	Transcription
	XP_015621184.1	La-related protein 1A isoform X1	VPDSQR	VPDS*QR	0.754	0.0000	13.6500	Transcription
	XP_015642930.1	Septum site-determining protein	AGFFSFFGG	AGFFS*FFGG	0.556	0.0000	14.8483	Transcription
	BAF08718.2	ATP-dependent DNA helicase	KFEHEPK	KFEHEPK		0.0000	16.1209	Translation
	BAS70391.1	E3 ubiquitin-protein ligase	DLLNATKR	DLLNATKR		0.0000	18.5204	Protein degradation
	XP_015640159.1	Arginyl-tRNA--protein transferase 2	QSSVNKNTVR	QSSVNKNTVR		0.0000	17.3091	Protein degradation
	XP_015641088.1	Peroxygenase 4	MASKPADVTATGGGGVAVVR	MAS*KPADVTATGGGGVAVVR	0.520	0.0000	17.6856	Metabolic process
**KDML105**	BAD53112.1	Chalcone synthase	QIGDSIGR	QIGDS*IGR	0.762	0.0000	20.6759	Defense response
	BAF29919.2	Phenylalanine ammonia-lyase	VDAAEAFR	VDAAEAFR		0.0000	17.6121	Defense response
	CAE03366.1	Osjnbb0065l13.9	AGMAVWMRR	AGMAVWMRR		0.0000	18.5368	Transcription
	CAE04537.2	Osjnba0040d17.5	YILSAPILKGR	YILSAPILKGR		0.0000	16.7900	Transcription
	CAE05823.1	Osjnba0028m15.15	VHKDYK	VHKDYK		0.0000	17.3996	Transcription
	ABF99104.1	Ribosomal protein s14p/S29e	NLSFFMADK	NLSFFMADK		0.0000	17.5785	Translation
	XP_015622394.1	UDP-N-acetylglucosamine O-acyltransferase	RLLIASR	RLLIASR		0.0000	18.1314	Metabolic process

**Table 2:** The increased DEPs in both rice cultivars in response to CTS induction with osmotic stress treatment.

Cultivar	Accession Number	Protein Name	Peptide	Peptide Sequence and Predicted Phosphorylation Site (*)	Score	Intensity		Functional Category
						CON+PEG	CTS+PEG	
**LPT123**	AAK13126.1	Histidine-kinase-like protein	AEVTMYHLR	AEVTMYHLR		0.0000	19.2691	Signal transduction
	AAX95871.1	Protein kinase domain	KVVEHNGK	KVVEHNGK		0.0000	13.8992	Signal transduction
	BAS86337.1	WD-domain-containing protein	LVIFDG	LVIFDG		0.0000	16.1930	Signal transduction
	BAF05127.1	Disease resistance protein RPM1	IGGMR	IGGMR		0.0000	17.5347	Defense response
	XP_015611347.1	ABC transporter C family member 4	SSLLGCILGEMR	SS*LLGCILGEMR	0.532	0.0000	20.2736	Transport
	XP_015612553.1	Cytochrome b561	SGDTSSR	SGDT*S*S*R	0.667, 0.984, 0.670	0.0000	17.7423	Transport
	BAF08853.2	DNA polymerase epsilon catalytic subunit	EEGVLLK	EEGVLLK		0.0000	16.4910	Replication
	AFI71858.1	Beta-amylase 1, chloroplastic	MSESGSPR	MS*ESGSPR	0.566	0.0000	16.3572	Metabolic process
	BAS93628.1	Chaperone protein dnaJ 49	LTKGMDGNK	LT*KGMDGNK	0.629	0.0000	16.7405	Protein folding
	BAS95271.1	T-complex protein	DPPVFLRI	DPPVFLRI		0.0000	16.4615	Protein folding
**KDML105**	XP_015617069.1	Receptor-like serine/threonine-protein kinase	TAQAK	T*AQAK	0.621	0.0000	16.2963	Signal transduction
	XP_015624189.1	B3 domain-containing protein Os02g0598200	TSNQNGEKNMK	T*SNQNGEKNMK	0.660	0.0000	18.3402	Signal transduction
	AAQ74383.1	Na/H antiporter	SLHSPLLTR	S*LHS*PLLT*R	0.567, 0.596, 0.524	0.0000	19.2654	Transport
	B9G2A8.1	Auxin transport protein BIG	KLGSSILSSR	KLGS*SILS*S*R	0.731, 0.853, 0.899	0.0000	19.2937	Transport
	XP_015642960.1	Membrane protein of ER body-like protein	AGLKVITIIDK	AGLKVITIIDK		0.0000	21.3910	Transport
	XP_015626888.1	Serine/arginine-rich SC35-like splicing factor SCL30	EHEVDK	EHEVDK		0.0000	20.8443	Transcription
	AAT77858.1	Translational activator	AILGGSEGK	AILGGSEGK		0.0000	18.8387	Translation
	ABA95184.1	Lipase family protein	DVLTLVTK	DVLT*LVTK	0.511	0.0000	16.2153	Metabolic process
**KDML105**	ABF96062.1	Branched-chain amino acid aminotransferase	RNSPNSIDSK	RNS*PNS*IDS*K	0.734, 0.985, 0.993	0.0000	19.0565	Metabolic process
	XP_015613842.1	U-box domain-containing protein 45	GSSCK	GSS*CK	0.810	0.0000	19.7904	Protein degradation
	XP_015627057.1	Ubiquitin carboxyl-terminal hydrolase 15	VEALKKPSK	VEALKKPS*K	0.980	0.0000	20.0381	Protein degradation
	XP_015651426.1	Polyadenylation specificity factor	HLGAAQIDR	HLGAAQIDR		0.0000	20.2841	Metabolic process

## Data Availability

All data are included in the main text.

## Supplementary Materials

The following supporting information can be downloaded at: https://www.mdpi.com/article/10.3390/plants11202729/s1, Table S1: phosphoproteins in LPT123; Table S2: phosphoproteins in KDML105.
